# Using an extended model of the reasoned action approach to explore individual behavioral intentions regarding litter and plastic pollution prevention in a developing country

**DOI:** 10.3389/fpsyg.2023.1274765

**Published:** 2024-01-10

**Authors:** Kwaku Oduro-Appiah, Abraham Afful, Henrietta Osei-Tutu

**Affiliations:** ^1^Department of Water and Sanitation, University of Cape Coast, Cape Coast, Ghana; ^2^Deutsche Gesellschaft für Internationale Zusammenarbeit (GIZ), GmbH, Accra, Ghana; ^3^Greater Accra Resilient and Integrated Development Project, Ministry of Sanitation and Water Resources, Accra, Ghana

**Keywords:** litter prevention, behavioral intentions, reasoned action approach, moral norms, attitudes, plastic pollution, marine litter, urban resilience

## Abstract

Implementing litter and plastic pollution prevention strategies is essential for cities of developing countries, mainly due to the prevailing high incidence of littering and the urgent need to realize the adverse *per capita* environmental impact target of the sustainable development goals. In this article, we report the use of the prominent reasoned action approach—in its original state and an extended model with moral norms—for exploring the critical socio-cognitive determinants of individuals’ litter prevention intentions in Ghana. By analyzing the valid answers of 447 participants to a structured questionnaire on litter prevention, we found attitudes (*β* = 0.35, SE = 0.014, *p* < 0.001) and moral norms (*β* = 0.57, SE = 0.099, *p* < 0.001) as the most influencing determinants to individual intentions in the original and the extended models, respectively. The analysis suggests that individuals will stop littering their environments if environmentally friendly interventions are implemented to elicit self-responsibility and moral obligation. Campaigns that demonstrate the effects of littering on drain blockage, flooding, and disease outbreaks may improve individual litter prevention attitudes. Installing waste receptacles in public spaces and communicating persuasive messages may facilitate personal antilittering intentions. Apart from contributing to the implementation of a litter management strategy to reduce the flood risk and enhance the resilience of the Greater Accra region of Ghana, this research helps to close the literature gaps in litter prevention behavior in developing countries, as well as support the implementation of the sustainable development goals and the global plastic action partnership.

## 1 Introduction

Starting in 2025, the world’s oceans will receive an estimated 17.5 million tonnes of mismanaged plastic waste annually from coastal cities around the globe (Jambeck et al., 2015). Rivers reportedly will contribute an average of 1.8 million tonnes of mismanaged plastics annually to the oceans via inland sources (Lebreton et al., 2017). This will affect marine life, the environment, public health and livelihoods (Jambeck et al., 2018; Agamuthu et al., 2019; Beaumont et al., 2019; Welden, 2020; Diggle and Walker, 2022). Estimates show that developing countries would contribute at least 80% of the mismanaged waste from land to the oceans (Schmidt et al., 2017). In most cities of such economies, the solid waste management (SWM) systems are highly underdeveloped, with low-to-medium collection coverage and waste capture (Guerrero et al., 2013; Wilson et al., 2015; Godfrey et al., 2019; Oduro-Appiah et al., 2020). Consequently, significant amounts of uncollected municipal solid waste (MSW) are disposed of in drains and on vacant lands, which are eventually washed down by stormwater into rivers and oceans (Godfrey et al., 2018).

Plastic leakage refers to the amount of plastic waste that is not properly managed and ends up in the environment (OECD, 2022). In 2020, Ghana was reported to have leaked about “80,000 tonnes of plastic waste into water bodies” (Global Plastic Action Partnership, 2021). Insufficient MSW collection coverage (Oduro-Appiah et al., 2020), inadequate disposal of plastic waste, microplastics and littering on land and along beaches are significant factors in plastic leakage (OECD, 2022). The leakage is projected to increase threefold within the next two decades, with an anticipated increase in economic growth and plastic consumption (Global Plastic Action Partnership, 2021). Reversing the increasing trends of plastic pollution in freshwater bodies and the growth of waste patches within the world’s oceans would call for modernization in municipal solid waste management (MSWM) and litter prevention, especially in developing country cities (Leal Filho et al., 2021).

Littering is the inappropriate discarding of any piece of solid waste (SW) in a public place outside designated trash receptacles; it is a persistent problem globally, with prominence in most developing countries (Tjell, 2010; Moqbel et al., 2020). In Ghana, littering is rampant and socially acceptable, as in some other developing countries of the global south (Farage et al., 2021). It has been identified as one of the main causes of perennial flooding, especially in the capital city, Accra, which has become a national cause of concern. Nonetheless, neither the national government nor MSW professionals seem to have sustainable policies to address the problem, the prevailing system being an end-of-pipe management technique where communities litter their environment before cleaning and evacuation.

Within the past decade, the closest the government of Ghana has come to solving the problem of littering and the leakage of plastic waste into freshwater and marine environments has been the introduction of a national sanitation day and a tax on some imported plastic materials (Adam et al., 2020); with the objectives to promote inclusive community cleaning of the environment and to decrease the consumption and leakage of single-use plastics, respectively. However, the outcomes have been more counterproductive and far from the goal (Abalo et al., 2017; Adam et al., 2020; Mensah, 2020), notwithstanding the enormous costs of such interventions to the state. It is thus not uncommon to see litter and uncollected MSW washed into drains, streams and rivers (Odonkor and Sallar, 2021), contributing to perennial flooding with loss of lives and properties, especially in the capital city, Accra (Wilson et al., 2015; Amoako and Inkoom, 2018; Mensah and Ahadzie, 2020).

One such flood, which occurred on June 3, 2015, in the greater Accra region of the country, had a devastating effect—over 50,000 inhabitants were displaced, along with the loss of 150 human lives and an estimated US$ 100 million in properties (Kpanou et al., 2021); compelling politicians and professionals alike to seek a comprehensive pathway concerning the implementation of an MSW collection improvement and a litter control strategy, as part of other interventions aimed at reducing the flood risk, enhancing the resilience of the region and preventing plastic leakage to the oceans (Government of Ghana, 2017; World Bank, 2019).

However, because litter prevention is considered a pro-environmental behavior (Homburg and Stolberg, 2006), understanding the principal determinants and values that inform individual litter prevention intentions may offer significant clues to support and sustain the development and implementation of evidence-based and theory-driven antilittering behavior change interventions (Gifford and Nilsson, 2014; Steg, 2016; Chaudhary et al., 2021).

The scholarly literature on littering and litter prevention recommends the provision of adequate trash receptacles, the promotion of environmental cleanliness, social norms, personal norms, penalties, incentives and the implementation of other social marketing tools to address littering behavior (Ojedokun, 2013; Moqbel et al., 2020; Chaudhary et al., 2021); however, the paucity of such literature on developing countries (Al-mosa et al., 2017a), alongside the varying outcomes of such interventions suggests that litter prevention beliefs and intentions may be context-specific (Weaver, 2015; Al-mosa et al., 2020), varying from place to place (Freije et al., 2019).

So far, only three studies on littering and litter prevention behavior in Ghana have been identified in the peer-reviewed literature. The first assessed the extent of litter pollution at four beaches along the coast in the greater Accra region through a survey (Van Dyck et al., 2016). Another explored the antecedents of littering behavior among University students through interviews (Amankwah-Poku and Ofori, 2020), and the third used a socio-cognitive behavior theory to predict household waste disposal, with an implied reference to littering (Tweneboah-Koduah et al., 2019).

This article uses an extended model of the reasoned action approach (RAA) to explore the predominant latent constructs and beliefs that may influence individual intentions about litter prevention in Ghana. The use of theory and especially the RAA for predicting and designing interventions targeted at reducing littering behavior is applicable in the scholarly literature (Brown et al., 2010; Al-mosa et al., 2017a; Chaudhary et al., 2021; Ojedokun et al., 2022). Being the first of its kind within the country, the objective is two-fold, namely: (1) to determine the influencing factors that may support practitioners to develop research-based litter prevention behavior change interventions for the region, and (2) to provide a baseline data that may support the implementation of the behavioral components of a national plastic action roadmap that seeks to reduce plastic leakage in Ghanaian waters and the oceans (Global Plastic Action Partnership, 2021). The research for this article is thus very important since it aims to identify the factors that impact people’s decisions to litter and dispose of waste (especially used plastics) in water bodies. Determining such socio-cognitive factors and using the same to design and disseminate communication and awareness campaign strategies engenders behavioral change in the targeted audience and leads to better pro-environmental outcomes.

The article builds further on the work of Tweneboah-Koduah et al. (2019), with a point of departure in two key areas, namely: (1) the use of formative research to elicit the relevant beliefs within the target population and (2) the addition of a fourth latent construct, moral norms, to extend the original RAA. Moral norms are reported to improve the predictive viability of pro-environmental behaviors such as litter prevention and waste separation at the source (Godin et al., 2005; Oduro-Appiah et al., 2022). On the other hand, formative research supports researchers to determine the prominent beliefs of the intended audience for the designing of applicable survey instruments (Downs and Hausenblas, 2005), thereby preventing them from using previous research items and predetermined ideas, both of which may lead to unreliable and unrealistic outcomes (Ajzen, 2015). The RAA was selected for this research because of its ability to support the prediction of pro-environmental behaviors (Yuriev et al., 2020; Cudjoe et al., 2022; Ojedokun et al., 2022) and its adaptability to the addition of new constructs to the original model (Miller, 2017).

## 2 Literature review and hypothesis formulation

### 2.1 The reasoned action approach

The reasoned action approach (RAA) is a theoretical model for the prediction of people’s intentions and behavior (Fishbein and Ajzen, 2011) and has been used extensively to predict several human social and pro-environmental behaviors (Hardeman et al., 2002; De Leeuw et al., 2015; Yuriev et al., 2020; Cudjoe et al., 2022). Examples include the use of the RAA to predict source separation behavior (Ma et al., 2018), recycling behavior (Strydom, 2018; Du and Pan, 2021), littering behavior (Ojedokun et al., 2022), energy-saving behavior (Obaidellah et al., 2019; Liu et al., 2020) and climate change mitigation and adaptation behaviors (Zhang et al., 2020), to name a few.

**Figure 1:**
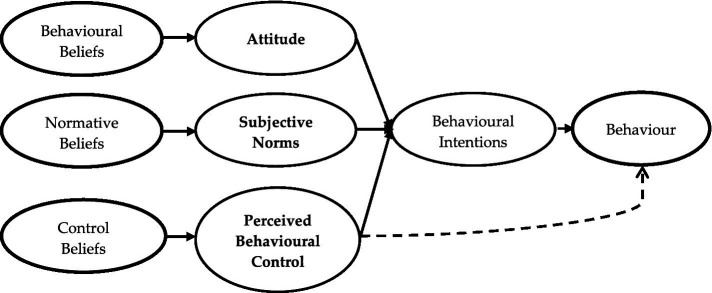
The original RAA framework (Fishbein and Ajzen, 2011).

Primarily, the RAA explains the relationships between individuals’ beliefs, attitudes, norms, behavior control, intentions and actual behavior. The theory postulates that behavioral intention is the immediate precursor to actual behavior (Ajzen, 1991) and that three sub-determinants of behavior, namely, attitudes, subjective norms, and perceived behavioral control (PBC), predict intentions (Figure 1).

The RAA defines intention as a person’s willingness to perform a behavior (Fishbein and Ajzen, 2011); attitude as a person’s assessment of the perceived favorable and unfavorable outcomes of performing a behavior; subjective norms as the perceived social pressure from significant others that influences an individual to act or not to perform a behavior, and perceived behavioral control (PBC) as the extent to which individuals perceive the behavior to be carried out to be under their control. According to the theory, PBC may also influence actual behavior directly (Bamberg and Möser, 2007) (see Figure 1).

According to the RAA, each of the three latent predictors of intention is determined from the multiplicative amalgamation of individual belief outcomes and the evaluations of the outcomes. Thus, attitude is drawn from behavioral belief outcomes, —which represent the individual’s beliefs of the consequences of performing the behavior— and the evaluations of the outcomes. Subjective norms are expressed from normative beliefs, —which represent the individual’s beliefs of what significant others expect them to do—, as well as the individual’s motivations to comply with such expectations, and PBC is drawn from control beliefs, —which represent the individuals’ beliefs of the factor (s) that could enable or hinder them from executing the behavior—, as well as their power to manage the behavior. The RAA reasons that individuals will undertake a behavior when (1) they perceive positive outcomes from it (2) they feel strong social pressure from significant others to perform the behavior, and (3) they are convinced that they can accomplish the behavior. However, it must be emphasized that individuals place different weights on each latent construct in estimating their preparedness to perform a behavior. The implication is that one or two latent variables may be more dominant than the others based on the individual’s perceptions, worldviews, knowledge, and experiences about the behavior to be performed (Klöckner, 2013).

In this study, individuals’ attitudes are described as the degree to which they expect favorable and unfavorable outcomes in preventing littering and plastic pollution. Where individuals perceive positive outcomes, they will likely stop littering. Subjective norms refer to the perceived influence from significant others that will compel an individual to prevent littering. In contrast, PBC refers to the individual’s perceived capability to stop littering. Thus, according to the RAA, it is expected that individuals would have strong intentions to stop littering when they perceive positive outcomes to be associated with litter prevention, believe significant others in their lives will expect them to stop littering, and when they are confident of themselves to have what it takes to stop littering.

**Figure 2:**
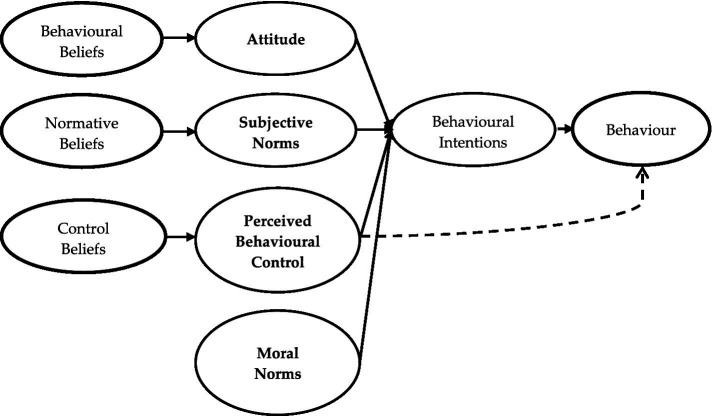
Extended RAA model with moral norms.

Moral norm was added as a fourth sub-determinant (Figure 2) to the original RAA model to assess its power to improve the predictive viability of individual litter prevention intentions. Moral norms are individual perceptions of the ethical appropriateness or inappropriateness of performing a particular behavior (McMahon and Byrne, 2008). Thus, relating it to this study, we defined moral norms as the individuals’ perceived principles about the ethical appropriateness of preventing the littering of the environment. Our choice of moral norm as an additional construct was influenced by recommendations in the scholarly literature of the use of a measure of moral norms to improve upon the predictive viability of individual’s intentions to engage in pro-environmental behaviors like recycling, source separation, litter and plastic pollution prevention, that has clear moral dimensions (Godin et al., 2005; Bamberg and Möser, 2007; Rivis et al., 2009; Fishbein and Ajzen, 2011; Klöckner, 2013; Rousta et al., 2020).

The RAA has also proven—throughout the years—to be adaptable to new latent constructs, and evidence abounds to the improvement in model performance and the variance in pro-environmental behavioral intentions upon such additions (Sandberg and Conner, 2008; Yuriev et al., 2020). For example, moral norm has been used as an additional construct in the RAA to predict: hotel guest energy-saving behavior (Wang et al., 2021), household source separation intentions (Razali et al., 2020; Oduro-Appiah et al., 2022), recycling intentions and behaviors (Tonglet et al., 2004; Botetzagias et al., 2015), food waste reduction and source separation behavior (Graham-Rowe et al., 2015; Yuan et al., 2016; Wang et al., 2022), and particulate matter reduction behavior (Ru et al., 2019).

Based on recommendations from the literature, we employed moral obligation, personal values, responsibility, environmental respect and feelings of guilt to measure moral norms (Tangney et al., 2007; Rivis et al., 2009; Oteng-Peprah et al., 2020; Rousta et al., 2020). Relating to this study, we defined moral obligation as the individual’s perception of whether it ethically right or wrong to stop littering; values as goals that serve as guiding principles for individuals to halt littering (Schwartz, 1992); responsibility as the individuals’ involvement in litter prevention programs because of their understanding and commitment to keep to their obligations (Moretto et al., 2011) and feelings of guilt as the self-conscious negative emotional state aroused in an individual for not participating in a litter prevention program (Tangney et al., 2007). Moral obligation, values, responsibility and emotions have been used to favorably predict moral norms and pro-environmental behaviors (Sandberg and Conner, 2008; Steg, 2016).

### 2.2 Hypothesis development

Even without extending the RAA with additional constructs, the three main original latent constructs [attitude, subjective norm, perceived behavioral control (PBC)] of the RAA have been found to successfully predict pro-environmental behavior through the mediating role of intentions (Botetzagias et al., 2015; Kumar, 2019; Liu et al., 2020). We thus propose in our first hypothesis that:

*H1*: Each of the original RAA latent constructs, attitude, subjective norm and PBC, positively predicts individuals’ behavioral intentions to stop littering and plastic pollution of the environment.

Due to the proven adaptability of the RAA to the inclusion of new explanatory constructs, several constructs (habits, personal norms, moral norms, past behavior, situational factors, etc.) have been used to expand the original RAA model in the prediction of pro-environmental behavioral intentions and actual behavior (Ghani et al., 2013; Botetzagias et al., 2015; De Leeuw et al., 2015; Miller, 2017; Oteng-Peprah et al., 2020). Amongst these, moral norm appears to be the most commonly used additional construct, and evidence abounds to the improvement in model performance and the variance in pro-environmental behavioral intentions upon such additions (Sandberg and Conner, 2008; Rousta et al., 2020; Yuriev et al., 2020). For example, moral norm has been used as an additional construct in the RAA to predict: hotel guest energy-saving behavior (Wang et al., 2021), household source separation intentions (Razali et al., 2020; Oduro-Appiah et al., 2022), recycling intentions and behaviors (Tonglet et al., 2004; Botetzagias et al., 2015), food waste reduction and source separation behavior (Graham-Rowe et al., 2015; Yuan et al., 2016; Wang et al., 2022), and particulate matter reduction behavior (Ru et al., 2019). Nonetheless, the use of moral norms to extend the RAA to determine litter prevention intentions in a developing country context is limited in the scholarly literature. Therefore, we propose that:

*H2*: Moral norms will positively predict individual behavioral intentions to stop littering and plastic pollution of the environment.

*H3*: Moral norms will directly improve the predictive viability of individuals’ littering and plastic pollution prevention intentions.

## 3 The study setting: the Odaw River catchment

**Figure 3:**
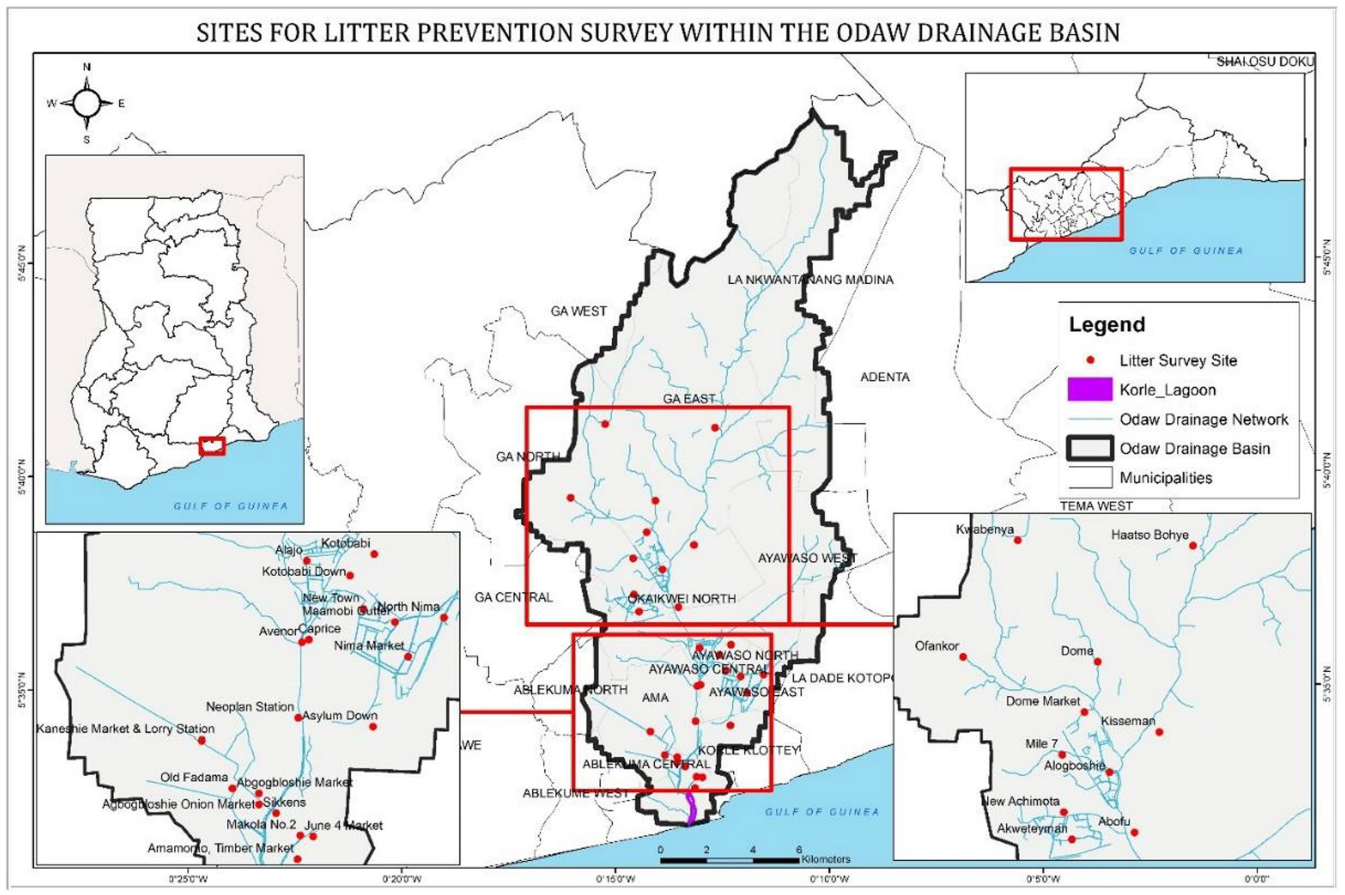
The Odaw River basin depicting communities where the litter prevention survey was conducted. Source: The authors.

The research for this article was conducted within the catchment of the Odaw River in Ghana. The river is about 30 km long and covers an estimated 270 km^2^. It is regarded as the most polluted catchment in the country (Amoako and Frimpong Boamah, 2015; Ackom et al., 2020), serving as both a water source and a solid waste sink (Ansa et al., 2017). The river originates from the country’s Eastern region and flows through about 70% of the greater Accra region, discharging into the Atlantic Ocean via the Korle Lagoon (see Figure 3). Inadequate MSWM, littering, and siltation contribute to the frequent flooding of the river and the region (Erman et al., 2020; Amaglo et al., 2022).

**Figure 4:**
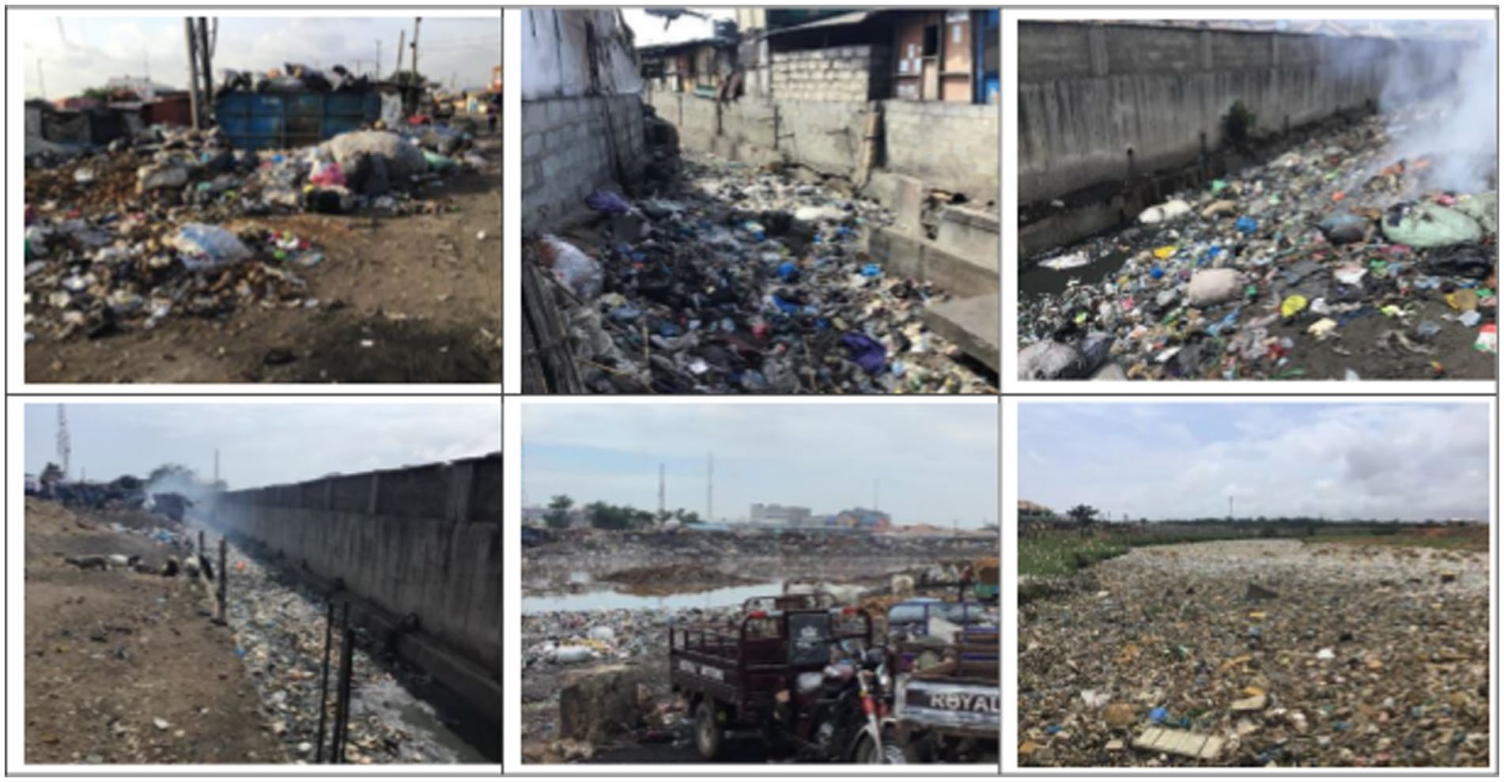
Extremely high incidence of littering within the Odaw River basin. Source: The authors.

The basin’s daily MSW generation rate is 2,240 tonnes, of which 37% is uncollected. Most inhabitants of dense settlements (houses and small businesses) along the entire stretch of the river dispose of waste directly into it (Ntajal et al., 2022). That, as well as wind-blown litter and run-off-washed MSW, account for about 410 tonnes of SW daily into the river. Littering is rampant within the basin (Figure 4), but the MSW handlers lack a sustainable prevention strategy. Apart from the government’s institutionalized National Sanitation Day, which is intended to clean the region of litter and MSW once every month, the ministry in charge of sanitation and water resources seldomly installs a few 240-liter plastic bins along some major roads within the central business districts to reduce the extent of littering and plastic pollution. However, such interventions have failed because of the lack of participatory planning processes between stakeholders. Paradoxically, the inability of system handlers to educate the inhabitants on the purpose of the waste receptacles and the absence of a sustainable waste removal plan has turned most of such locations into temporal disposal sites, with an extremely high incidence of littering. We selected the catchment for this study to support ongoing project interventions that seek to strengthen the region’s resilience through littering and plastic leakage prevention, flood risk reduction, and MSW collection service delivery improvements (Government of Ghana, 2017; World Bank, 2019; Global Plastic Action Partnership, 2021).

## 4 Materials and methods

### 4.1 Design, sample description and procedure

The study was conducted as part of a data collection exercise to support the development of a regional MSW collection improvement and litter management strategy. Structured questionnaires were administered continuously by two of the authors and five trained investigators for 8 days in October 2020. The questionnaire was categorized into two: one on individual demographics and the other on the extended latent variables. Based on recommendations from the framers of the RAA, belief items elicited in a formative study were used indirectly to measure the original RAA constructs (Ajzen, 2002; Fishbein and Ajzen, 2011). In total, 451 randomly selected individuals from 22 communities participated in the research (Figure 3). The communities represented the region’s three socio-economic divides: low-, middle- and high-income. The target participants were approached in their homes and environs and encouraged to participate in the survey voluntarily. Computer-assisted personal interviewing was used to capture participants’ responses and monitor the questionnaire administration process in real time.

### 4.2 Formative research

Formative research is a critical requirement of the RAA (Ajzen, 2015). It supports the use of the predominant beliefs of the target audience in developing survey instruments rather than the prearranged beliefs of researchers (Downs and Hausenblas, 2005). We conducted the formative research for this study by administering beliefs-related questions to 30 participants selected randomly within the target population (Ajzen, 2002; Fishbein and Ajzen, 2011). According to the RAA, individuals’ behavior is influenced indirectly by behavioral-, normative- and control beliefs concerning the behavior and the evaluations of the corresponding outcomes (Fishbein and Ajzen, 2011). Behavioral beliefs were elicited by asking the participants to write down or mention three anticipated merits and demerits of litter prevention to their environment. Normative beliefs were obtained by asking the participants to list separately the significant others who would endorse or disapprove of them stopping littering. Finally, control beliefs were elicited by asking the participants to record at least three factors that would make it easy or difficult for them to stop littering. Before asking the questions, the investigators explained the concept of littering, litter prevention and plastic leakage prevention to all participants. The three most prominent behavioral, normative and control beliefs, each within the population, were gleaned—after a content analysis of the responses using Microsoft Excel.

### 4.3 Materials, measures and pretesting for the main study

We used the prominent belief outcomes from the formative research to prepare the questionnaires for the study. Based on the recommendations of the proponents of the RAA, we measured the original RAA sub-determinants with belief outcomes and their evaluations (Fishbein and Ajzen, 2011). In contrast, the fourth sub-determinant, moral norms and the dependent determinant, intention, were measured with only belief outcomes. Attitudes, subjective norms and perceived behavioral control were each measured with three belief items. Moral norms and behavioral intention were measured with five and two belief items, respectively. All belief items and their outcome evaluations were measured as statements on a 5-point Likert scale. The validity and consistency of the questionnaire were improved upon after pretesting them on 50 individuals outside the drainage basin. The final questionnaires were modified to suit its objectives based in part on the recommendations of a social psychologist and after the computation of correlation coefficients.

#### 4.3.1 Measurement of the attitude construct

Behavioral belief outcomes related to the prevention of drain blockage, disease and flooding were used to assess participants’ beliefs about the consequence of stopping littering. Among the three behavioral outcomes list, participants were asked to rate the likelihood that preventing littering would produce each outcome on the 5-point Likert scale ranging from “extremely bad” to “extremely good.” For example, on the behavioral belief outcome “drainage blockage,” participants were asked to choose on the scale if “preventing drainage blockage” was considered to be “extremely bad” or “extremely good.” They were then asked to rate the importance of each outcome (i.e., the belief strength) on a 5-point Likert scale from “highly unlikely” to “highly unlikely.” Among the belief strengths were “I will stop littering to prevent the blocking of drains by waste materials,” “I will stop littering to prevent the outbreak of disease,” and “I will stop littering to prevent the occurrence of floods” The total behavioral belief score was computed according to the expectancy-value model by multiplying each behavioral outcome evaluation by the corresponding belief strength and summing all together (Ajzen and Fishbein, 2008). The total of each normative and control belief measure was computed based on the multiplicative combination of subjective probabilities and values.

#### 4.3.2 Measurement of the subjective norm construct

Concerning subjective norms, “neighbors, family members and community leaders (obtained as outcomes from the formative research) were used as the significant others to measure the normative beliefs of participants. Participants were asked to indicate —on the 5-point Likert scale—the extent to which the three significant others would expect them to stop littering their environment and their motivation to comply with such expectations. For example, on subjective belief strength, participants were asked to rate on a scale ranging from “highly unlikely” to highly likely” whether their neighbor will expect them to stop littering for the good of the environment. Participants were then asked to rate on the scale ranging from “definitely not” to “yes, definitely” their motivation to comply with the question “For issues relating to litter prevention, what my neighbor think I should do is what I will do.

#### 4.3.3 Measurement of the perceived behavioral construct

In contrast, the provision of waste bins at vantage points, educational campaigns on the effects of littering on public health and the environment, and enforcement of penalties were used as the control beliefs to assess participants’ perception of the factors that would make it easy for them to stop littering. Regarding the perceived power of control in the belief, participants were allowed to indicate their preference on a 5-point Likert scale ranging from “definitely not” to “yes definitely” whether “having access to waste receptacles,” being educated on the effects of littering on the environment,” and punishing culprits of littering through the enforcement of penalties” will enable, make it easy and encourage them to stop littering. The control belief strength was measured by asking participants to indicate on a scale ranging from “highly unlikely” to “highly likely if they will stop littering should they have access to receptacles, see evidence of culprits being penalized and be provided with the requisite know-how.

#### 4.3.4 Measurement of the moral norm construct

Participants were asked to indicate their preference on the 5-point Likert scale ranging from “definitely not” to “yes, definitely” the following questions as a direct measure of moral norms, namely: “I consider it as doing something morally right if I stop littering the environment,” “For me, my values prevents me from littering the environment” “For me, I consider it as a sense of responsibility to stop littering for the good of the environment,” “I consider it as respect for the environment to stop littering” and “I will feel guilty if I do not stop littering.

#### 4.3.5 Measurement of the intention to stop littering

Participants were asked to indicate their preference on the 5-point Likert scale ranging from “definitely not” to “yes, definitely” and “not sure” to “surely,” respectively, to the following questions as a direct measure of behavioral intentions, namely: “I plan to stop the littering of the environment,” and I intend to stop littering the environment.

### 4.4 Analysis of data and fit indices

There were no missing data, but four responses were identified as outliers after the screening, leaving 447 valid responses for confirmatory analysis. Editions 24 of both SPSS and AMOS were used for the data analysis. Based on recommendations, we recoded the Likert scale range of 1 to 5 of all belief outcomes to −2 to +2 (Ajzen and Fishbein, 2008; Fishbein and Ajzen, 2011). The original and the extended RAA models were then fitted to the data using structural equation modeling. Because the data were normally distributed, the models were run after performing confirmatory factor analysis using the maximum likelihood parameter estimation.

The scale measuring the constructs was also improved by deleting two belief items with low loading factors: “enforcement of penalties” and “feeling guilty.” As recommended, we evaluated both the original and the extended RAA models using the root mean square error of approximation (RMSEA), the normed fit index (NFI), the Tucker-Lewis Index (TLI), the comparative fit index (CFI), the incremental fit index (IFI) and the goodness of fit index (GFI) (Schreiber et al., 2006). Generally, NFI, TLI, CFI, IFI, and GFI values between 0.90 and 0.95 and RMSEA values between 0.06 and 0.08 are good fits. RMSEA is expected to be lower than 0.06 for excellent fits, with the other indices recording more than 0.95.

## 5 Results

### 5.1 Descriptive statistics

The demographics of the survey participants are in Table 1. The total number of valid responses was 447, with 60% females and 40% males. The mean age was 40 years, and about 85% of respondents had completed primary school through to tertiary. Participants exhibited positive intentions, favorable attitudes, moderate social norms, moderate controllability and high moral norms in preventing littering of their environment (Table 2). All the constructs correlated significantly to stop littering.

**Table 1:** Demographic details of the participants.

Variable	Category	Frequency	Percent
1. Gender	Male	179	40
	Female	268	60
2. Educational level	No education	67	15
	Primary school	54	12.1
	Junior high school	149	33.3
	Senior high school	122	27.3
	Technical/Vocational certificate	11	2.5
	Technical/Vocational diploma	13	2.9
	Tertiary school	31	6.9

**Table 2:** Summary of covariances and means.

	Cronbach’s alpha	Mean	*S.D*	*A*	*B*	*C*	*D*	*E*
*A*. Attitude^1^	0.87	7.79	0.42	1				
*B*. Subjective norms^1^	0.67	5.11	1.17	0.34**	1			
*C*. Perceived Control^1^	0.55	4.14	0.75	0.11**	0.38**	1		
*D*. Moral norms^2^	0.80	4.38	0.08	0.43**	0.57**	0.17**	1	
*E*. Intention^2^	0.84	4.51	0.01	0.20**	0.15**	0.31**	0.50**	1

^**^p < 0.05, Theoretical range (−10–10)^1^, (1–5)^2^.

### 5.2 Analysis of the original model

**Figure 5:**
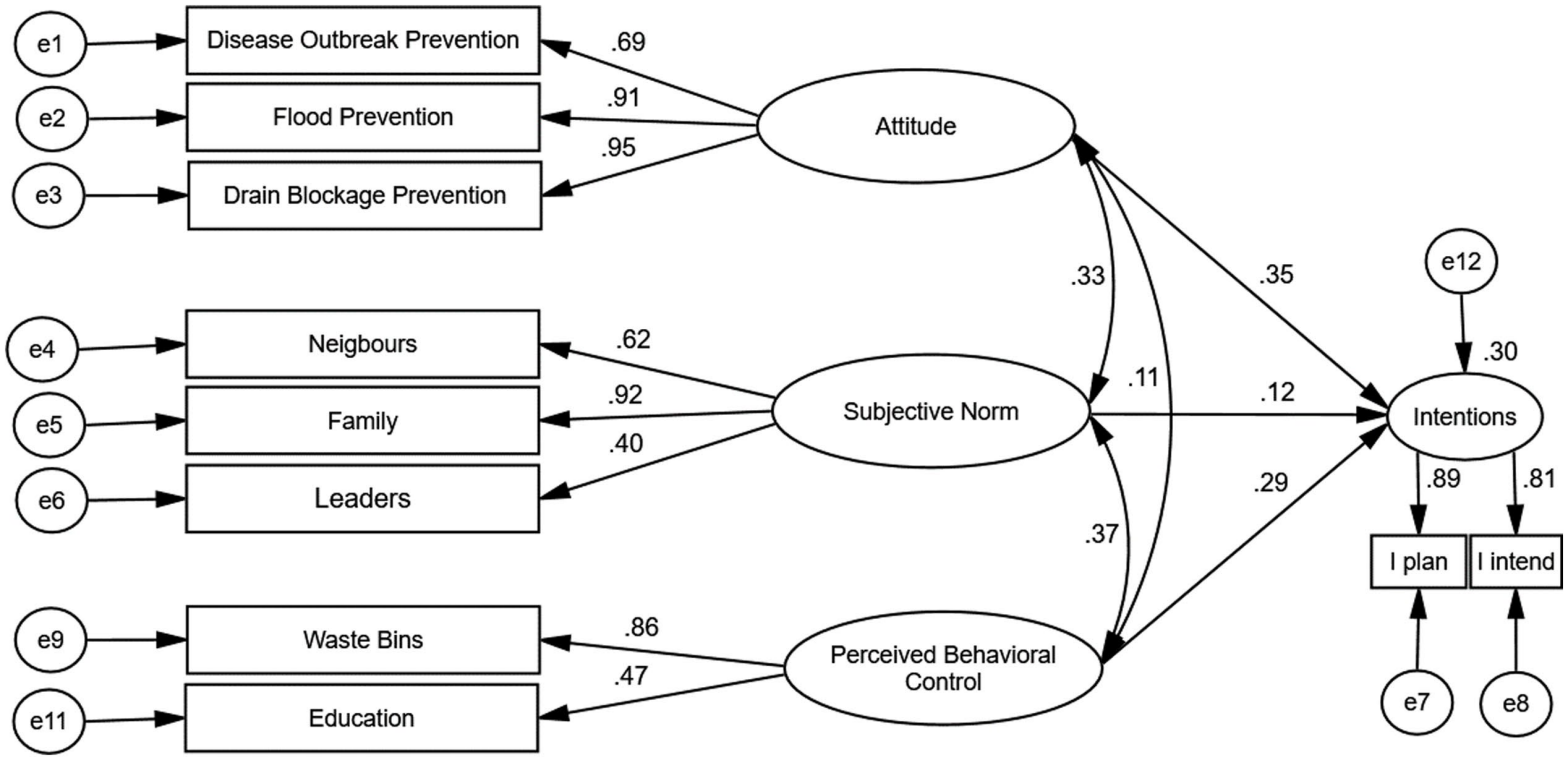
The original model.

The original structural equation model depicting individuals’ intention to stop littering within the catchment is displayed in Figure 5. The arrows depict the presumed direction of causal influence, while the numerical values beside each single-headed arrow depict the standardized path (regression) coefficients. The doubled-head arrows depict the correlations between the latent constructs. The model fitted the data adequately (RMSEA = 0.073, NFI = 0.95, TLI = 0.94, CFI = 0.96, IFI =0.96, IFI = 0.96, GFI = 0.96) and accounted for 30% of the variance in participants behavioral intentions. Attitude (*β* = 0.35, SE = 0.014, *p* < 0.001) appeared as the strongest predictor of participants’ intention to stop littering, followed by perceived behavioral control (*β* = 0.29, SE = 0.020, *p* < 0.05). The subjective norm construct (*β* = 0.12, SE =0.012, *p* = 0.64) could not significantly predict individual intentions to stop littering. The prevention of drain blockage (*λ* = 0.95), flooding (*λ* = 0.91) and disease outbreaks (*λ* = 0.69) came up as the salient behavioral beliefs to influence litter prevention within the catchment (Figure 5).

### 5.3 Analysis of the extended model

**Figure 6:**
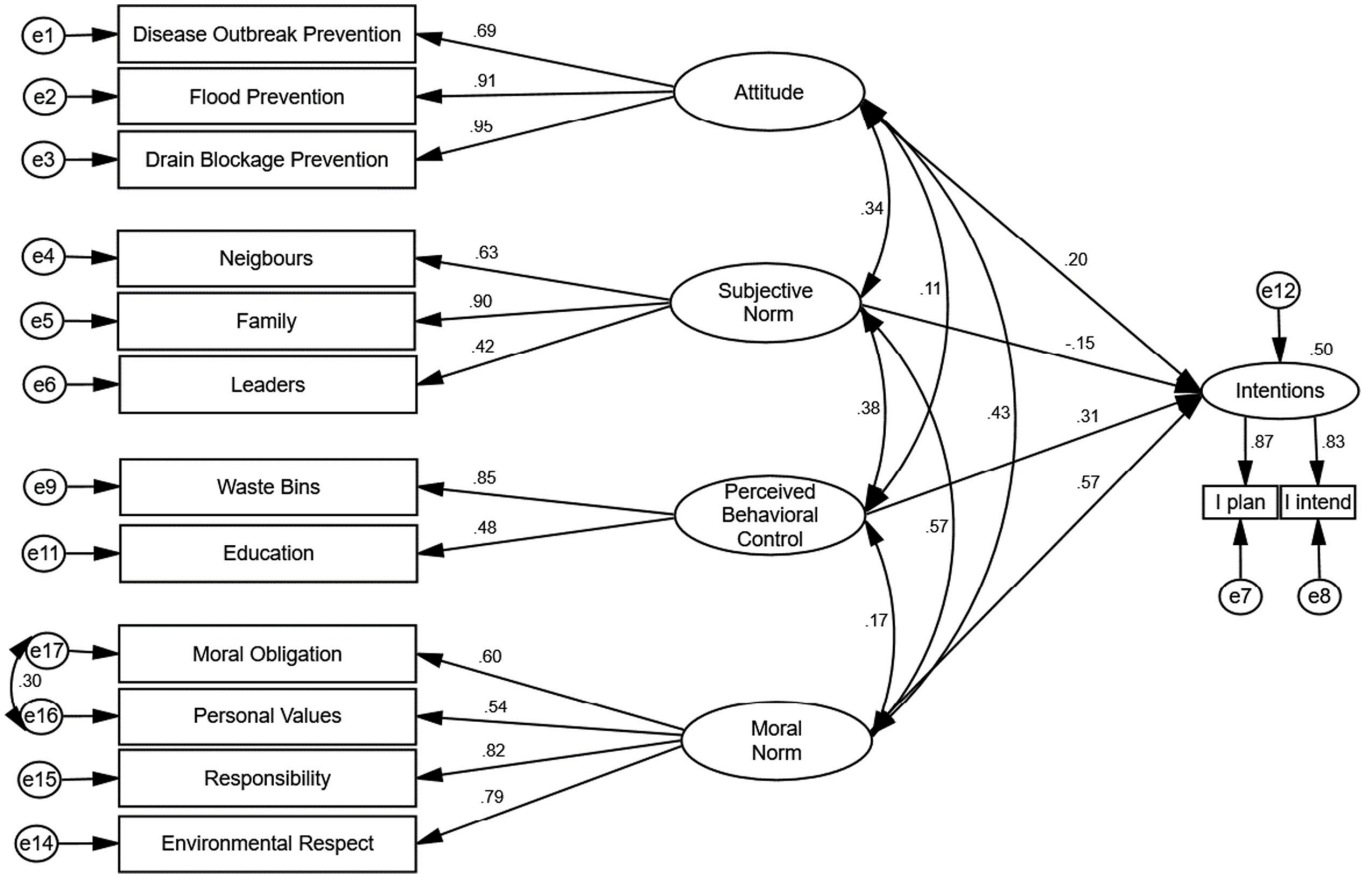
The extended model.

Figure 6 is the extended model with moral norms as the additional determinant of intentions. This model improved the prediction of intentions by explaining 50% of the variance in behavioral intentions to stop littering, as well as providing an excellent fit to the data (RMSEA = 0.06, NFI = 0.94, TLI = 0.94, CFI = 0.96, CFI = 0.96, IFI = 0.96 and GFI = 0.95). The corresponding factor loadings (also shown in Figure 6) and *t*-values are shown in Table 3. Moral norms (*β* = 0.57, SE = 0.099, *p* < 0.001) emerged as the most vital construct to positively influence the behavioral intentions of participants about litter prevention, followed by perceived behavioral control (*β* = 0.31, SE = 0.013, *p* < 0.001) and then attitudes (*β* = 0.20, SE = 0.014, *p* < 0.001). However, the subjective norm construct (*β* = −0.15, SE = 0.014, *p* = 0.03) was statistically significant but negatively predicted behavioral intentions in the extended model. Overall, we found moral norms, perceived behavioral control and attitude to be the predominant predictors of individuals’ intentions to stop littering. Individual beliefs related to the provision of waste bins (*λ* = 0.85), individual responsibility (*λ* = 0.82), environmental respect (*λ* = 0.79) and moral obligation (*λ* = 0.60) appeared to be the most influential in stopping littering in the region (Figure 6).

**Table 3:** Summary of factor loadings and *t*-values.

Beliefs	Construct	Estimate	Factor loadings	*t*-values
Disease outbreak prevention	Attitude	0.71	0.69	17.87
Flood prevention	Attitude	0.94	0.91	28.62
Drain blockage prevention	Attitude	1	0.95	–
Neighbor	Subjective norm	0.56	0.63	10.68
Family	Subjective norm	1	0.90	–
Leaders	Subjective norm	0.38	0.42	7.72
Waste bins	PBC	1	0.85	–
Education	PBC	0.37	0.48	4.87
Moral obligation	Moral norm	0.72	0.60	12.18
Personal values	Moral norm	0.61	0.54	10.72
Sense of responsibility	Moral norm	1	0.82	–
Showing respect	Moral norm	0.97	0.79	16.00

## 6 Discussions

The prediction of behavioral intentions and the design of effective behavior change interventions require a considerable amount of planning and research (Ajzen, 2015). This study used the RAA to predict the predominant latent constructs and understand the most readily accessible beliefs about litter prevention intentions in the greater Accra region of Ghana. A theory provides a foundational platform to identify the complex relationships between constructs and the development of evidence-based behavioral change interventions (Glanz and Bishop, 2010; Conner and Norman, 2015; Ojedokun et al., 2022).

In this research, two structural models were used to test the hypothesis: (1) the original model with the three RAA constructs as the predictors of individuals’ litter prevention intentions (Figure 5) and (2) an extended model where moral norms were added as a fourth sub-determinant to the original model (Figure 6). The findings indicate that the intention to stop littering and plastic pollution of the environment was significantly predicted by two of the original RAA latent constructs (attitudes and PBC) but not with subjective norms, consistent with earlier findings in which subjective norms were found to be a weak predictor of litter prevention intentions (Carmi et al., 2015; Tweneboah-Koduah et al., 2019).

### 6.1 Theoretical implications

In the original model, attitudes emerged as the most influential construct to individual behavioral intentions about litter prevention in the region, consistent with findings on waste disposal and litter prevention behaviors in Ghana and other developing countries (Tweneboah-Koduah et al., 2019; Ibrahim et al., 2021; Singh and Kaur, 2021). In the extended model, moral norms appeared as the dominant construct influencing individual intentions, supporting the second hypothesis (H2) in which we proposed that moral norms will positively influence the individual’s behavioral intention to stop littering the environment. The outcome is also similar to findings on pro-environmental behaviors and litter prevention in developing countries, in which moral and personal norms were reported to positively affect individuals’ antilittering intentions and behavior (Gifford and Nilsson, 2014; Moqbel et al., 2020; Farage et al., 2021).

We found moral norms, perceived behavioral control and attitudes as the three most significant constructs influencing individual litter prevention intentions in Ghana. The relatively strong prevalence of the perceived behavioral construct in both models is also supported in the literature (Ojedokun et al., 2022). More often than not, limited financing, low levels of spending and the absence of user inclusivity strategies in the MSWM systems of most developing countries (Wilson et al., 2013; Oduro-Appiah et al., 2017; Kanhai et al., 2021) restrict residents’ access to waste receptacles and educational campaigns on sustainable MSW practices, affecting their motivation and ability to perform pro-environmental behaviors.

The original model fitted the data adequately and explained 30% of the variance in individuals’ intentions to stop littering. The extended model provided an excellent fit to the data, explaining 50% of the variance in intentions. In conformity with literature and expectations, adding the moral norm construct improved the RAA’s predictive power in both the explained variance and the fit indices (Walsh et al., 2005; Yuriev et al., 2020), supporting the third hypothesis (H3), in which we proposed that the addition of moral norm was to improve upon the predictive viability in behavioral intentions to stop littering. However, subjective norms significantly but negatively predicted intentions in the extended structural model. Plausible reasons could be the influence of the moral norm construct in the extended RAA. Research on similar pro-environmental behaviors such as recycling has identified the dominant influence of moral personal norms over social norms (Botetzagias et al., 2015), in which individuals intentions to perform pro-environmental behaviors that have clear moral dimensions, like litter prevention, are greatly influenced by moral personal norms rather than societal pressures (Schwartz, 1977). For example, it has been found that parents’ and peers’ subjective norms indirectly influence adolescents’ pro-environmental behaviors through personal norms (Collado et al., 2017). Another reason may be the adoption of only injunctive norms in measuring subjective norms. Evidence abounds of individuals’ willingness to perform pro-environmental behaviors based on descriptive norms (what other significant others are doing concerning the behavior) rather than what the significant others expect them to do (White et al., 2009; de Leeuw et al., 2014). These same reasons may be attributed to the high correlation coefficient between subjective and moral norms within the extended structural model.

The use of formative research contributed to understanding the readily accessible individual beliefs about litter prevention in the region. We found positive behavioral belief outcomes related to the prevention of drain blockage, flooding and disease outbreaks as the most prominent to influence individual attitudes toward litter prevention. Control beliefs on individuals’ accessibility to waste receptacles appeared as the most influential in encouraging participants to stop littering, consistent with earlier findings in Ghana in which accessibility, time and convenience to locating waste bins are reported to improve individual waste disposal behaviors (Tweneboah-Koduah et al., 2019). Even though subjective norms negatively influenced individual intentions, families and neighbors emerged as the most influential referents to compel individuals to stop littering, similar to results in other developing countries like India and Nigeria (Singh and Kaur, 2021; Ojedokun et al., 2022). Evaluating the beliefs that influence moral norms points to individual responsibility, environmental respect and moral obligation as the most influential factors, consistent with similar findings in developing country cities (Moqbel et al., 2020; Farage et al., 2021).

### 6.2 Practical implications

What the findings mean to litter and plastic pollution prevention and MSW modernization policy in the catchment, the country, and perhaps similar lower-middle-income cities is that decision-makers must implement a comprehensive litter management strategy that integrates social-psychological and technical factors toward litter prevention (Ojedokun, 2011). MSW system handlers may have to work with communication professionals to roll out educational campaigns to establish the linkages between litter prevention and reducing the incidence of waste-related drain blockage, flooding and disease outbreaks. This should be done alongside investment in locally appropriate trash receptacles in all public places within the catchment to persuade inhabitants to dispose of plastic and other MSW in an eco-friendly manner (De Kort et al., 2008; Kombiok and Naa Jaaga, 2023). System handlers should design the trash receptacles to allow users to practice at least a two-stream waste separation of biodegradables and all others (plastics, metals, paper) to promote recycling and divert organic and recyclable waste from landfills (Oduro-Appiah et al., 2022). Because the extent of cleanliness of an environment is reported to be positively associated with litter prevention behavior (Rangoni and Jager, 2017; Al-mosa et al., 2017b), implementing a sustainable waste collection strategy with targeted and improved collection coverage may further prevent littering around centrally placed receptacles. The total effect of moral norms on litter prevention intentions suggests that promoting litter and plastic pollution prevention is a morally right thing to do when it comes to MSW management and protecting water bodies.

The considerable extent of the littering problem in the region and other developing country cities calls for the use of inclusive governance approaches to engage all stakeholders —policy makers, funding institutions, researchers, waste experts, social psychologists, communication strategists, MSW professionals, environmentalists, solid waste service providers, informal waste service and value chain actors and especially the citizens— to systematically implement and monitor interventions. That, in addition to using persuasive and demonstrative messages that hinge on individual self-responsibility, moral obligation and respect for the environment, would create ownership of interventions and empower the inhabitants to stop littering (Cingolani et al., 2016).

## 7 Limitations and recommendations

The study used a cross-sectional methodological approach and could not measure actual behavior, partly because litter prevention has yet to be officially and systematically promoted and practiced in the region. Neither is there evidence of the presence and availability of the required infrastructural and social-psychological investments that will encourage individuals to stop littering. Although a recent study in Nigeria (Ojedokun et al., 2022) positively correlated litter prevention intentions to actual behavior, we recommend a further longitudinal study during the implementation of interventions to study the relationship between behavioral intentions and actual conduct.

Secondly, the current study was limited to only individuals within the Odaw River basin of Ghana. It may thus restrict the generalizability of the findings to the country and beyond. We recommend similar studies in different regions to support system handlers to understand better the complex mix of latent variables and beliefs concerning litter prevention in the country. Thirdly, we adopted only the component of injunctive norm to measure subjective norm. In hindsight and based on the influence of the subjective norm construct on litter prevention intention, we recommend any study on littering in the country to measure the influence of descriptive norm on intentions if the RAA is the theoretical framework. Fourthly, the original latent constructs of the RAA were not directly measured to allow for comparison between the indirect and direct measures. Further research using other behavioral theories, such as the focus theory of normative conduct and the norm activation model, may help stakeholders compare the research outcomes to sustain the implementation of interventions.

## 8 Conclusion

The study’s main objective was to use an extended model of the reasoned action approach (RAA) to predict the social psychological determinants and understand the prominent beliefs that influence individuals’ litter prevention intentions in the greater Accra metropolitan area of Ghana. The study constitutes part of interventions to develop a litter management strategy to reduce flooding, prevent plastic leakage and enhance the region’s resilience. Using structural equation modeling, two models were evaluated–the original RAA model and an extended model in which moral norm was added as a fourth latent construct to the original model. The original model adequately fitted the data and explained 30% of the variance in intentions. The extended model improved the predictability of behavioral intentions with an excellent fit to the data and explained 50% of the variance in litter prevention intentions. We found moral norms (*β* = 0.57, SE = 0.099, *p* < 0.001), perceived behavioral control (*β* = 0.31, SE = 0.013, *p* < 0.001) and attitudes (*β* = 0.20, SE = 0.014, *p* < 0.001) as the predominant constructs that influence individual intentions concerning litter prevention in the region. Subjective norms failed to predict intentions significantly in both models. Well-organized pro-environmental campaigns that promote self-responsibility (*λ* = 0.82), environmental respect (*λ* = 0.79), and moral obligation (*λ* = 0.60) present the most significant opportunity to support litter prevention within the region. Educational campaigns that establish the linkage between littering and drain blockage (*λ* = 0.95), flooding (*λ* = 0.91) and disease outbreaks (*λ* = 0.69) may improve individual attitudes about antilittering. In contrast, the provision of waste receptacles at public places and vantage points (*λ* = 0.85), supported by the publication of persuasive messages via the electronic, print and social media platforms, may likely empower individuals to dispose of solid waste in an environmentally friendly manner. System handlers are encouraged to include the citizenry during project implementation to increase participation in the litter prevention process. Rewarding clean communities may improve upon subjective norms. Apart from contributing to narrowing the literature gap in littering and litter prevention in emerging economies, this research provides sustainable pathways for researchers and policymakers to address their MSW and littering problems concerning the attainment of clean cities.

## Data availability statement

The raw data supporting the conclusions of this article will be made available by the authors, without undue reservation.

## Ethics statement

Ethical review and approval was not required for the study on human participants in accordance with the local legislation and institutional requirements. Written informed consent from the participants was not required to participate in this study in accordance with the national legislation and the institutional requirements.

## Author contributions

KO-A: Conceptualization, Investigation, Methodology, Resources, Validation, Writing – original draft, Writing – review & editing. AA: Data curation, Formal analysis, Investigation, Methodology, Software, Validation, Writing – review & editing. HO-T: Data curation, Investigation, Methodology, Project administration, Resources, Validation, Writing – review & editing.
